# Autism Spectrum Disorder in a Single Family: A Case Series of Three Siblings

**DOI:** 10.7759/cureus.60362

**Published:** 2024-05-15

**Authors:** Madeha Kamal, Amna Ahmed, Abdulqadir J Nashwan

**Affiliations:** 1 Pediatrics Department, Sidra Medicine, Doha, QAT; 2 Nursing Department, Hamad Medical Corporation, Doha, QAT

**Keywords:** autism screening, neurodevelopmental conditions, chromosomal abnormalities, consanguineous marriages, autism spectrum disorder

## Abstract

Autism spectrum disorder (ASD) is a complex neurodevelopmental condition with a rising prevalence worldwide. While genetic factors are significantly associated with the disorder, environmental factors are often speculated to contribute to its onset. The Middle East, exhibiting higher rates of ASD, also sees frequent consanguineous marriages, necessitating focused studies on potential etiological factors in the region.

We report a unique case of a family with three children diagnosed with ASD. The parents, aged between 35 and 39 years at the birth of their first child, have no notable familial history of neurodevelopmental disorders. Interestingly, while both parents and two of the children had normal chromosomal patterns, one child displayed chromosomal abnormalities. This discrepancy raises questions about the interplay between genetics and external factors in the manifestation of ASD. The family’s medical history, combined with the regional context of high ASD prevalence and consanguineous marriages, provides a compelling backdrop for the study.

The presence of chromosomal abnormalities in only one child, despite no detectable genetic irregularities in parents or siblings, underscores the potential influence of environmental factors in the development of ASD. This case accentuates the importance of conducting in-depth genetic and environmental studies to unravel the intricate etiological web surrounding ASD in the Middle East.

## Introduction

Autism spectrum disorder (ASD) encompasses a range of neurodevelopmental disorders marked by challenges in social interaction and communication, along with repetitive behaviors and limited interests. Furthermore, individuals with ASD often experience abnormalities in sensory processing [1]. According to a 2019 World Health Organization report, it is estimated that 1 in 160 children globally have ASD, underscoring its prevalence [2]. In the United States, the rate of ASD diagnoses has seen a notable increase, rising from 1 in 59 children in 2014 to 1 in 54 children in 2020 [3]. The prevailing theories attribute this surge primarily to heightened awareness and more frequent diagnoses rather than a genuine increase in ASD cases. Notably, the countries with the highest autism prevalence, exceeding 100 per 10,000 healthy children, are all located in the Middle East, where Qatar has one of the highest rates at 120 per 10,000 healthy children compared to its small population [4].

The exact causes and mechanisms behind ASD remain elusive. However, it is generally accepted that a combination of genetic and environmental factors influences the onset of ASD, even if the specifics warrant further research [5]. In the realm of genetics, over 1,000 genes have been identified in relation to ASD. Yet only 25-30% of children diagnosed with ASD exhibit detectable ASD-related genes. This suggests that for nearly 70% of cases, non-genetic factors may be more dominant in their development [6]. As a result, the role of environmental influences in the emergence of ASD is undeniably significant.

## Case presentation

A mother with three children diagnosed with autism was interviewed, and this history was corroborated by examining the family's medical records. The mother was recently diagnosed with a benign ovarian tumor, while the father had hypertension and a family history of early coronary artery disease.

Their first child, a girl, was born when the mother was 35 years old. The birth was a normal vaginal delivery, and there were no complications necessitating admission to the neonatal intensive care unit. By age three, when the girl started attending daycare, the parents began noticing her challenges in social interactions and communication with her peers. They observed her lack of eye contact, repetitive movements, and social difficulties. After seeking medical advice, she was diagnosed with autism.

Their second child, a boy, was born when the mother was 38 years old. His birth was also uncomplicated. Early on, the parents recognized developmental concerns, such as the boy not responding to his name, avoiding eye contact, being engrossed in his own world, and displaying repetitive behaviors. By age 1.5 years, he was also diagnosed with autism.

**Figure 1:**
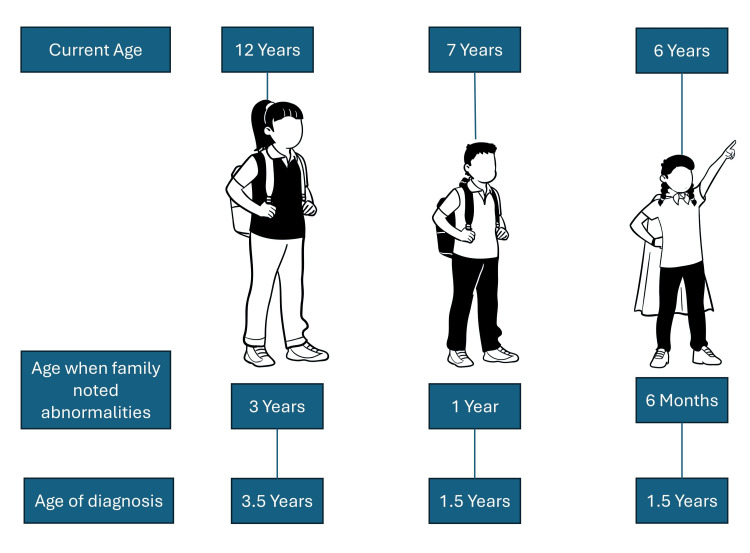
Developmental milestones timeline of the three children This figure has been created by Abdulqadir Nashwan, a co-author of this article.

The third child, a girl, was born when the mother was 39 years old. By the age of six months, the parents felt she was quite detached, raising concerns about potential autism. As she grew older, the signs became more evident: she would not respond to her name, avoided eye contact, had repetitive movements, followed strict routines, and showed sensory sensitivities. By the age of 1.5 years, she was diagnosed with autism as well (Figure 1). The family had a comprehensive understanding of autism, including repetitive movements, speech delays, lack of social interaction with parents or other children, and heightened sensitivity to loud voices or noises.

Given that all three children were diagnosed with autism, and the parents had no other children without the diagnosis, a chromosomal microarray was performed on the entire family. The results for both parents and two of the children were normal. However, the youngest girl's microarray analysis identified a two-copy gain of approximately 171 kilobases in the long arm of chromosome 1, specifically in the cytogenetic band 1q43. This change might affect the EDARADD gene on one or both alleles. Mutations in the EDARADD gene are linked to Ectodermal Dysplasia 11A, a condition related to hair and tooth anomalies. Notably, the girl did not display any symptoms of this condition.

The entire family is now participating in a research study involving Whole Exome Sequencing (WES) as part of the investigative process. Currently, the three children, ages 12, 7, and 6, are monitored in a developmental clinic.

## Discussion

ASD is a neurodevelopmental condition marked by challenges in social communication, as well as restricted interests and repetitive behaviors [7]. The current diagnostic criteria for ASD rely on the Diagnostic and Statistical Manual of Mental Disorders-5th edition (DSM-5), an evolution from its predecessor edition. In recent years, there has been a noted surge in autism diagnoses. Among the top five countries with autism rates surpassing 100 per 10,000 healthy children, Qatar has the highest prevalence at 151.2 per 10,000, equating to 1 in 66 individuals [8]. The prevailing understanding of ASD attributes it to a complex interplay between genetic and environmental factors, with heritability estimates varying between 40 and 80% [9].

In the presented cases, all three children met the ASD criteria as defined by the DSM-5, each manifesting distinct features and severity levels of autism. A study by Parner et al. identified 9556 children diagnosed with ASD and found associations between parental age and increased ASD risk in offspring. Specifically, both maternal and paternal ages were linked to heightened ASD risk, with hazard ratios spanning from 1.21 (1.10-1.34) to 1.65 (1.09-2.48), based on various age group combinations: under 35, 35-39, and 40+ years. For mothers under 35 years, ASD risk in their children rose with the father's increasing age, and conversely, for fathers under 35 years, the risk grew with the mother's age [10]. In this case, both parents were between 35-39 years old during the birth of their first child. It is well documented that advancing maternal age enhances the likelihood of chromosomal irregularities and trinucleotide repeat expansion in ova [9]. Intriguingly, in this case series, only one child exhibited chromosomal abnormalities. Consequently, a comprehensive research approach is being undertaken, encompassing advanced genetic testing such as WES, alongside examinations of potential environmental contributors, including nutritional and hormonal factors. According to Parner and his colleagues, ASD results from a complex interplay between genetic and environmental factors. In our case, all three children have autism despite only one having chromosomal abnormalities, which raises more questions about the effects of environmental causes and the overlap between genetic and environmental factors.

## Conclusions

This case series highlighted the need for deeper exploration into the causes of ASD within our society, particularly given the elevated prevalence rates observed in the Middle East. Considering the region's high incidence of consanguineous marriages, there's a pressing need for comprehensive genetic studies. However, environmental factors must not be overlooked. Interestingly, in our present study, only one child exhibited chromosomal abnormalities, even though both parents and the other two siblings yielded normal results. This further emphasizes the imperative to investigate environmental contributors to autism.
